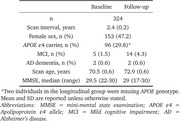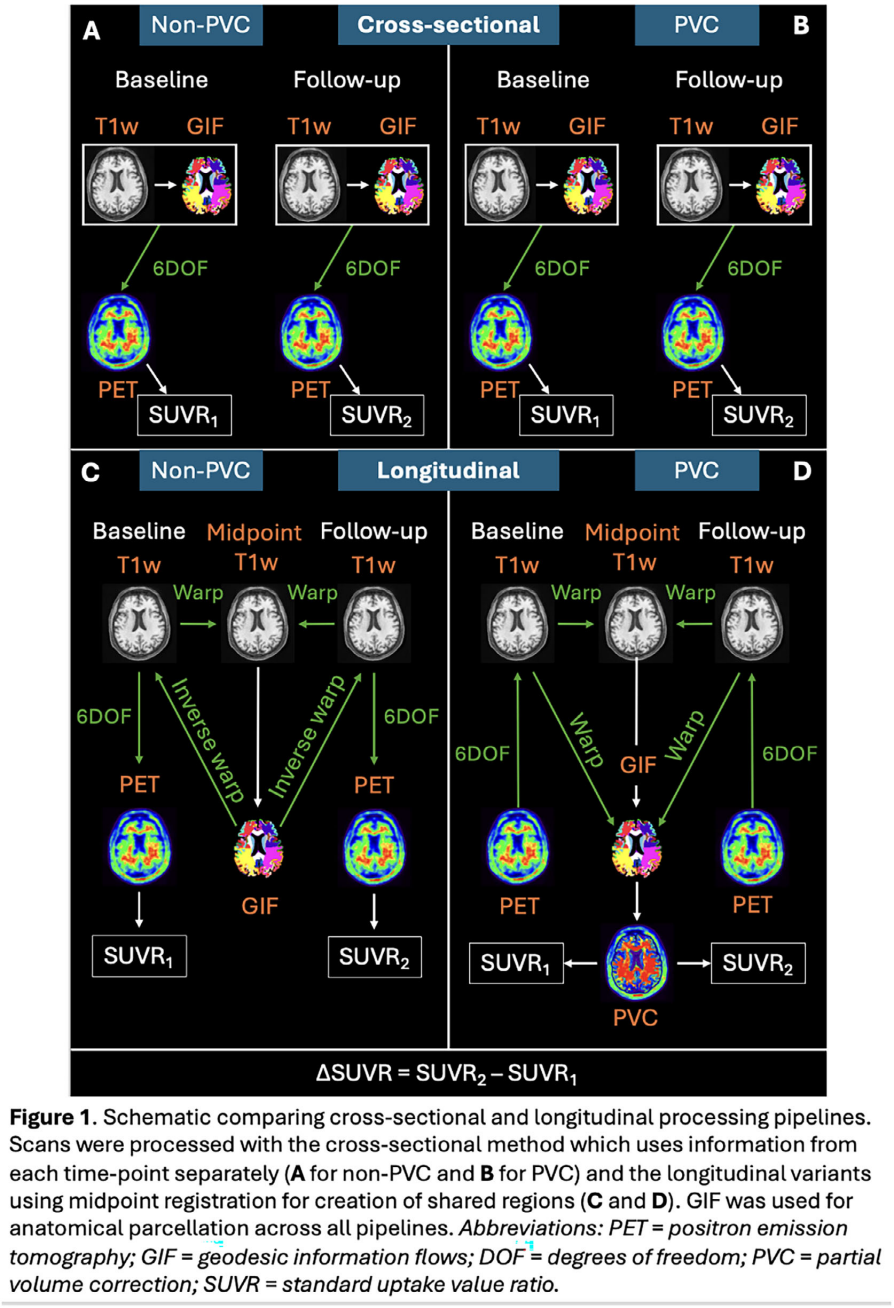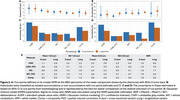# Improving longitudinal Aβ PET precision in the 1946 British birth cohort through anatomical consistency

**DOI:** 10.1002/alz70861_108753

**Published:** 2025-12-23

**Authors:** William Coath, Ariane Bollack, Catherine J Scott, Ian B. Malone, David L Thomas, Pawel J Markiewicz, Kjell Erlandsson, Frederik Barkhof, John Dickson, Michael Schöll, Jonathan M Schott, David M Cash, The Insight 46 team

**Affiliations:** ^1^ Dementia Research Centre, UCL Queen Square Institute of Neurology, University College London, London UK; ^2^ UCL Centre for Medical Image Computing, London UK; ^3^ Institute of Nuclear Medicine, University College London Hospitals, London UK; ^4^ Dementia Research Centre, UCL Queen Square Institute of Neurology, London UK; ^5^ Dementia Research Centre, Department of Neurodegenerative Disease, UCL Queen Square Institute of Neurology, University College London, London UK; ^6^ UCL Institute of Nuclear Medicine, London UK; ^7^ Department of Computer Science and Centre for Medical Image Computing, University College London, London UK; ^8^ University of Gothenburg, Gothenburg, Västra Götalands län Sweden; ^9^ Wallenberg Centre for Molecular and Translational Medicine and the Department of Psychiatry and Neurochemistry, University of Gothenburg, Göteborg Sweden; ^10^ UK Dementia Research Institute at UCL, London UK

## Abstract

**Background:**

Measuring Aβ change over time in cognitively unimpaired individuals is fundamental for preventative AD trials. The precision of longitudinal PET measurements may be enhanced through consistent anatomical labelling. Here we assessed the precision of cross‐sectional and longitudinal processing pipelines in a predominantly cognitively normal cohort.

**Method:**

Participants enrolled in Insight 46 (1946 British birth cohort) underwent combined PET/MRI with [^18^F]florbetapir at two timepoints (*n* =324, interval=2.4±0.2yrs, Table 1). We compared four different processing pipelines with anatomical regions defined on T1w‐MRI. Figure 1 shows each pipeline variant, the cross‐sectional pipeline used the T1w‐MRI from each time‐point separately, whereas the longitudinal pipeline first created a subject‐specific midpoint T1w‐MRI (using SPM12), each with/without iterative‐Yang partial volume correction (PVC). Standard uptake value ratios (SUVRs) from each of the pipelines were computed with five reference regions: cerebellar grey matter (CGM), whole cerebellum (WC), subcortical white matter (WM), pons and a composite. SUVRs were converted to centiloids (CL) and annual rate of change (ARC) calculated. Reliable detection of Aβ accumulation was assessed using Gaussian mixture modelling (GMM) with the best fitting model from 1‐3 Gaussians assessed using Bayesian information criterion. Bimodal models were used to define reliable accumulation status as ARC>99th percentile of the lower component. Bootstrapping was used to calculate 95% CI for cut‐points and reliable accumulator status. GMM parameters were used to calculate a signal‐to‐noise ratio (SNR) for each method.

**Result:**

ARC measured with WM and pons references, and the composite with PVC, did not show evidence of the expected bimodal distribution, instead best fitting a unimodal distribution, and were excluded from analysis. ARC from cross‐sectional and longitudinal pipelines were highly correlated for non‐PVC methods (rho>0.95, all reference regions) and slightly less correlated when PVC was used (CGM_PVC=0.80 and WC_PVC=0.88). Cut‐point estimates fell between 3.2 (WC_PVC, longitudinal) and 6.2 (CGM, cross‐sectional) CL/year. Precision of cut‐points and accumulator rate estimates were improved when using longitudinal processing with CGM_PVC and WC_PVC measures (Figure 2). Longitudinal processing boosted SNR substantially with CGM_PVC (cross‐sectional=1.0, longitudinal=1.9) and WC_PVC (cross‐ sectional=1.9, longitudinal=2.5; Figure 2E).

**Conclusion:**

Longitudinally consistent anatomical region definitions enhance precision in estimating change when using cerebellum‐based reference regions with PVC.